# Current Status of the Health Information Technology Industry in China from the China Hospital Information Network Conference: Cross-sectional Study of Participating Companies

**DOI:** 10.2196/33600

**Published:** 2022-01-11

**Authors:** Zhongan Zhang, Xu Zheng, Kai An, Yunfan He, Tong Wang, Ruizhu Zhou, Qilin Zheng, Mingfu Nuo, Jun Liang, Jianbo Lei

**Affiliations:** 1 Department of Information Center Affiliated Qingdao Central Hospital Qingdao University Qingdao China; 2 Peking University Third Hospital Beijing China; 3 Center for Medical Informatics Health Science Center Peking University Beijing China; 4 School of Public Health Zhejiang University Hangzhou China; 5 Department of Medical Informatics School of Public Health Jilin University Changchun China; 6 Department of Applied Mathematics Northwestern Polytechnical University Xian China; 7 Department of Bioinformatics and Biostatistics School of Life Sciences and Biotechnology Shanghai Jiao Tong University Shanghai China; 8 Institute of Medical Technology Health Science Center Peking University Beijing China; 9 Information Technology Center Second Affiliated Hospital School of Medicine, Zhejiang University Hangzhou China; 10 Key Laboratory of Cancer Prevention and Intervention, China National Ministry of Education School of Medicine Zhejiang University Hangzhou China; 11 School of Medical Informatics and Engineering Southwest Medical University Luzhou China

**Keywords:** medical informatics, China Hospital Information Network Conference, industry analysis, county medical community, smart hospital, cross-sectional study, digital therapeutic, information network, health care, hospital information, medical information, tertiary hospital

## Abstract

**Background:**

The China Hospital Information Network Conference (CHINC) is one of the most influential academic and technical exchange activities in medical informatics and medical informatization in China. It collects frontier ideas in medical information and has an important reference value for the analysis of China's medical information industry development.

**Objective:**

This study summarizes the current situation and future development of China's medical information industry and provides a future reference for China and abroad in the future by analyzing the characteristics of CHINC exhibitors in 2021.

**Methods:**

The list of enterprises and participating keywords were obtained from the official website of CHINC. Basic characteristics of the enterprises, industrial fields, applied technologies, company concepts, and other information were collected from the TianYanCha website and the VBDATA company library. Descriptive analysis was used to analyze the collected data, and we summarized the future development directions.

**Results:**

A total of 205 enterprises officially participated in the exhibition. Most of the enterprises were newly founded, of which 61.9% (127/205) were founded in the past 10 years. The majority of these enterprises were from first-tier cities, and 79.02% (162/205) were from Beijing, Zhejiang, Guangdong, Shanghai, and Jiangsu Provinces. The median registered capital is 16.67 million RMB (about US $2.61 million), and there are 35 (72.2%) enterprises with a registered capital of more than 100 million RMB (about US $15.68 million), 17 (8.3%) of which are already listed. A total of 126 enterprises were found in the VBDATA company library, of which 39 (30.9%) are information technology vendors and 57 (45.2%) are application technology vendors. In addition, 16 of the 57 (28%) use artificial intelligence technology. Smart medicine and internet hospitals were the focus of the enterprises participating in this conference.

**Conclusions:**

China's tertiary hospital informatization has basically completed the construction of the primary stage. The average grade of hospital electronic medical records exceeds grade 3, and 78.13% of the provinces have reached grade 3 or above. The characteristics are as follows: On the one hand, China's medical information industry is focusing on the construction of smart hospitals, including intelligent systems supporting doctors' scientific research, diagnosis-related group intelligent operation systems, and office automation systems supporting hospital management, single-disease clinical decision support systems assisting doctors' clinical care, and intelligent internet of things for logistics. On the other hand, the construction of a compact county medical community is becoming a new focus of enterprises under the guidance of practical needs and national policies to improve the quality of grassroots health services. In addition, whole-course management and digital therapy will also become a new hotspot in the future.

## Introduction

With the Chinese government's strong push for health care reform in 2009, the informatization construction of China's tertiary hospitals has basically completed the primary stage of popularization. The Chinese government first proposed taking health information technology (HIT) as the key direction for motivating medical reform in March 2009 [1] and vigorously promoted electronic medical records (EMRs). After 20 years of construction, the informatization of Chinese hospitals has made phased achievements. The Hospital Management Research Institute of the National Health Commission issued the new edition of evaluation criteria and management measures, which divided the application level of the EMR system into 9 levels ranging from 0 to 8 in December 2018. The Chinese government required that all tertiary hospitals reach grade 3 or above by the end of 2019 and that all tertiary hospitals reach grade 4 or above, while secondary hospitals reach grade 3 or above by the end of 2020 [2]. In 2019, 7870 medical institutions completed the graded evaluation of the application level of the EMR system, and the average level was 2.08. A total of 1874 tertiary hospitals participated in the evaluation, with an overall participation rate of 99.36%, and the average level exceeded grade 3 [3]. In addition, 34% of tertiary hospitals and 24.3% of secondary hospitals received level 5 or above. There were 0 institutions that received level 8, 4 institutions that received level 7, 19 institutions that received level 6, and 100 institutions that received level 5 [4]. It can be said that China's hospital informatization construction has completed the infrastructure construction stipulated by the National Health Commission and is facing the initial stage of digital transformation. This year, the China Hospital Information Network Conference (CHINC) 2021 was held during this special period.

The purpose of the conference is mainly communication, and it is also the most important way to understand the current situation of a country's industry. Conferences on medical informatization can be divided into two categories: academic and industrial. The most famous academic conference is the American Medical Informatics Association (AMIA) annual symposium. In 2020, more than 2100 people attended the online conference, involving 111 academic topics [5]. In addition, the most famous industrial conference is the Healthcare Information and Management Systems Society (HIMSS) conference, with more than 45,000 participants and 1300 enterprises. There are 4 well-known conferences in China, including two academic conferences (the Chinese Medicine Information Association Annual Symposium [CMIAAS] and the China Proceedings of Medical Informatics [CPMI]), and two industrial conferences (CHINC and the China Health Information Technology Exchange Conference [CHITEC]) [6]. The scale of China’s medical informatization academic conferences is small, with fewer than 1000 participants. Studies have shown that medical informatics conferences in China and the United States have differences and similarities. From the scale point of view, as mentioned above, even the largest CHINC in China has only half the number of participants as the HIMSS. From the perspective of discussion themes, EMRs are the research hotspot and focus shared by medical informatics academia and industry worldwide [7]. In contrast, China is more application oriented: the implementation rate of EMRs in Chinese hospitals has been approaching and surpassing that of the United States in recent years [8], but theoretical research and educational discussions are advanced in the United States [9].

In contrast, the scale of industrial conferences is much larger. CHITEC lasted for 2 days, and the number of participants reached 230,000 in 2020. CHINC introduced in this paper has a larger scale, a longer duration, and more submeetings compared with CHITEC.

Chinese hospitals are in the transition period of informatization and digitization. Understanding the research direction in the next stage is of great guiding significance for developing the medical information field. Therefore, CHINC, which involves many cutting-edge ideas, plays a special role in the field of medical information at this stage. CHINC is sponsored by the Institute of Hospital Management of the National Health Commission and has been held annually in China since 1995. It is one of the most influential academic and technical exchange activities in the field of medical informatics and medical informatization in China [6]. At present, it has successfully been held 25 times [10]. With the country's increasing attention toward public health and intelligent medical care, CHINC has attracted increasing attention. Only 6000 people attended conferences in 2016, but 17,000 people attended conferences in 2020. In addition, the number reached approximately 40,000 in 2021. Moreover, 2021 is the first year of the 14th 5-year plan. To implement the new requirements of the 14th 5-year plan and the Healthy China strategy for hospital construction and development, more than 400 experts gave wonderful lectures in 69 forums and academic activities, and 207 cooperative enterprises held roadshows to exchange and discuss new technologies, new achievements, and new experiences in hospital information construction to help the high-quality development of hospitals [11].

The main characteristics of CHINC include the organizer, history, cycle, holding time, number of participants, participating manufacturers, and conference forum. The number of participating enterprises and the main business of the enterprises are important factors reflecting the current situation of the industry. Therefore, we extracted the features of the enterprises participating in CHINC 2021. We analyzed the main concerns of the enterprises, gained insight into the current situation of China's medical information industry, and defined the future development direction. The conclusion can be used for reference by relevant experts in China and abroad.

## Methods

### Data Collection

**Figure 1:**
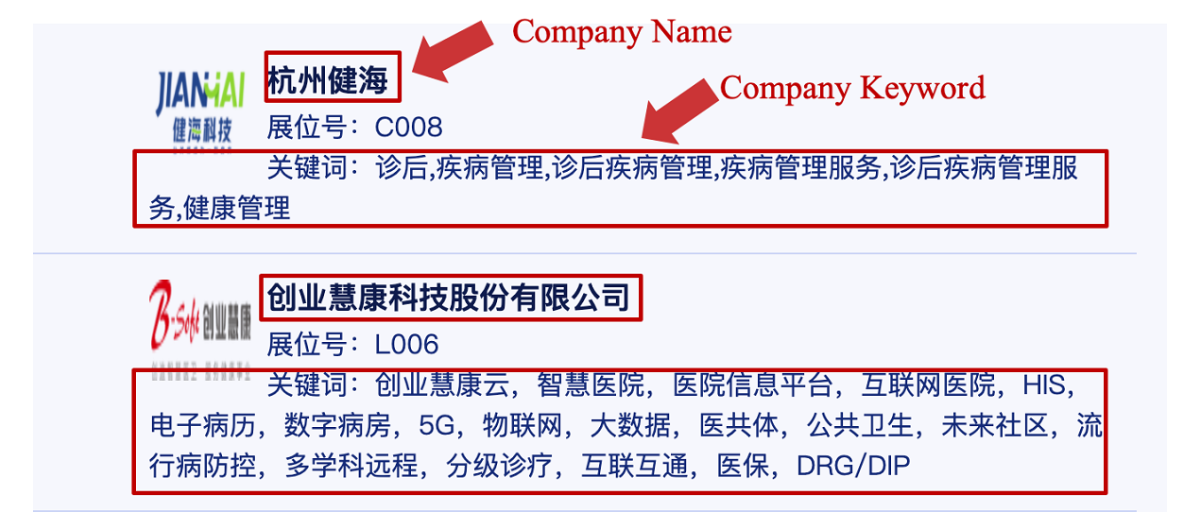
Information about enterprises provided on the official CHINC website. CHINC: China Hospital Information Network Conference.

First, the list of all participating enterprises (including enterprise name, exhibition booth, and keywords; Figure 1) was obtained from the conference’s official website [10]. We compared the list with the on-site list on the participation day one by one to exclude enterprises that did not attend the conference. Second, we used the TianYanCha website [12] to search all exhibitors and obtain basic information about the enterprises, including a brief introduction, region, establishment time, personnel scale, financing rounds, registered capital, and listing. Finally, we used the VBDATA company library [13] to obtain deep-seated information, such as the industrial field, application technology, and company concept of each enterprise. All data collection was completed by May 6, 2021.

### Data Storage and Analysis

We used Microsoft Excel 2019 for data storage and analysis. Percentages, bar charts, Venn charts, and statistical charts were used to display the exhibitors’ basic information, industrial and commercial information, classification, and grade data. Percentiles, medians, and quartile ranges were used to describe skew continuity data. We analyzed the data results according to the actual development of China's medical information industry.

## Results

### Exhibitors’ Characteristics

**Figure 2:**
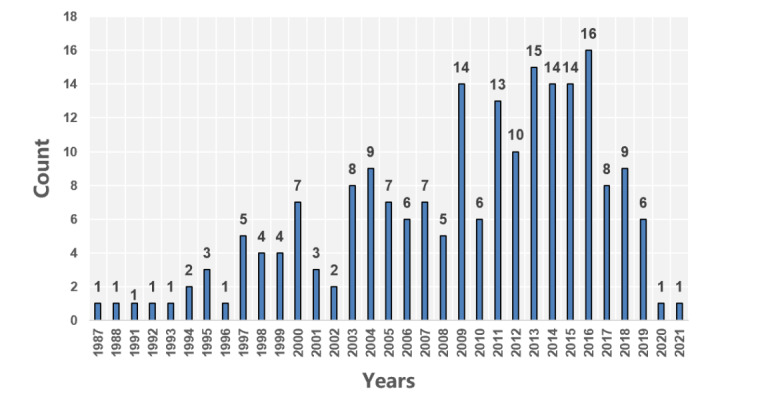
Establishment year of enterprises, displayed by year.

**Figure 3:**
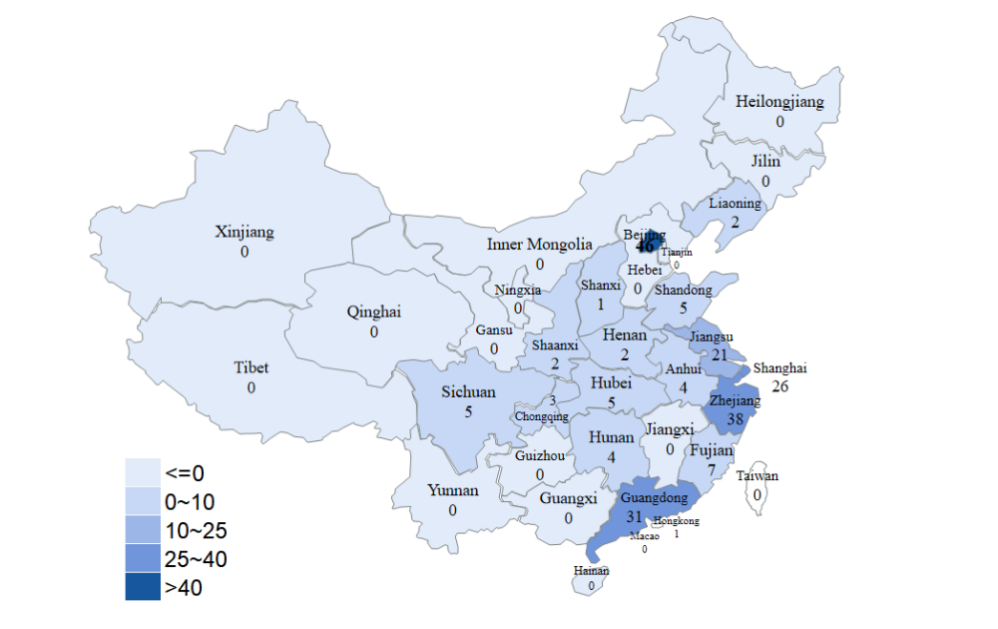
Establishment location of enterprises, displayed by province.

**Figure 4:**
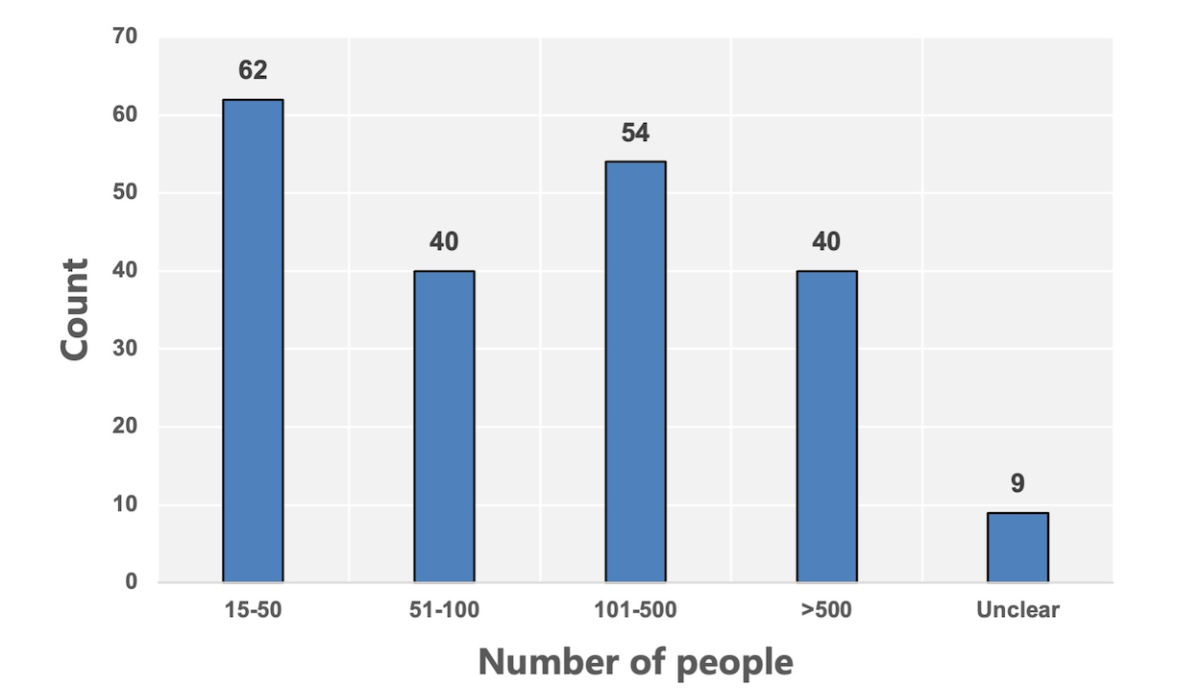
Establishment size of enterprises, displayed by scale.

A total of 207 cooperative enterprises were listed on the official website of CHINC. After checking each enterprise one by one on the conference day, the results indicated that 205 enterprises attended the conference. The participating enterprises were established from 1987 to 2021. A total of 61.9% (127/205) of enterprises were established from 2009, and 43.3% (55/127) of them were established from 2015 (Figure 2). A total of 79.02% (162/205) of the enterprises were from Beijing, Zhejiang, Guangdong, Shanghai, and Jiangsu Provinces, and 2 of them were from New Zealand (Figure 3). The enterprises’ scale is shown in Figure 4. Most enterprises (62/205, 30.02%) have fewer than 50 members, followed by 54/205 (34%) enterprises with 101-500 members.

### Industrial and Commercial Information Analysis

**Figure 5:**
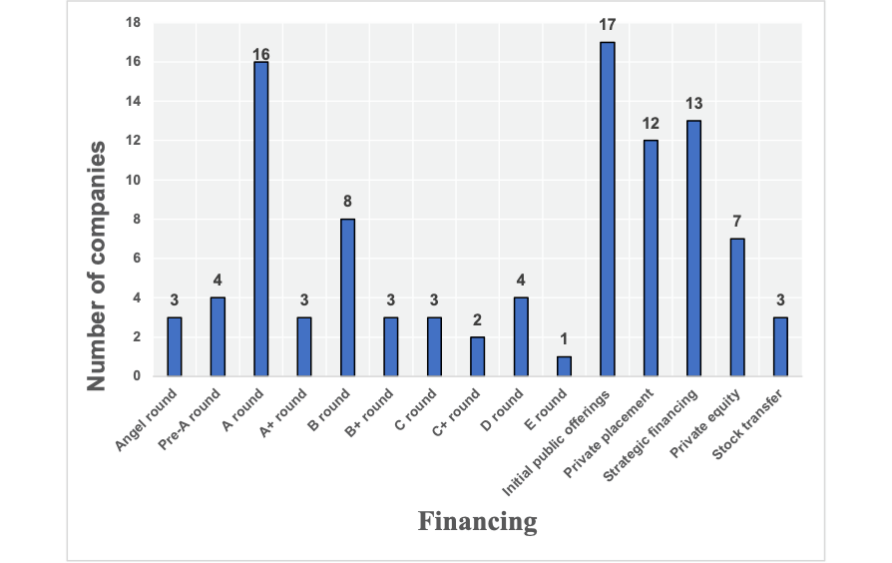
Financing of enterprises.

The median registered capital of the 205 enterprises participating in the conference is 16.67 million RMB (a currency exchange rate of RMB 1=US $0.16 is applicable; IQR 10-60 million RMB, maximum value=30 billion RMB; Table 1). In addition, 35 (17.1%) enterprises have a registered capital of more than 100 million RMB; see Table 2. Furthermore, 99 (48.3%) enterprises provided their financing information, of which 17 (8.3%) were initial public offerings (Figure 5).

**Table 1:** Registered capital of enterprises (N=205).

Variable	Minimum	P5^a^	P25^a^	P50^a^	P75^a^	P95^a^	Maximum
Enterprise registered capital (10,000 RMB^b^)	100	200	1000	1667	6000	86,941.15	30,000,000

^a^Px: Percentile occupied by the x-th position.

^b^A currency exchange rate of RMB 1=US $0.16 is applicable.

**Table 2:** Information about enterprises with more than 100 million RMB^a^ of registered capital.

Company name	Brief introduction	Area	Date of establishment	Registered capital/10,000 RMB	Scale, n
Hangzhou Century Co., Ltd.	Smart health care service provider	Zhejiang	November 21, 2003	86,941.15	>500
DHC MediWay Technology Co., Ltd	IT service provider in the big health field	Beijing	May 1, 2012	13,000.00	101-500
B-Soft Co.,Ltd	Hospital information platform provider	Zhejiang	December 10, 1997	110,962.71	>500
Beijing Lenovo Wisdom Medical Information Technology Co., Ltd	Integrated smart medical solution provider	Beijing	February 6, 2016	19,246.86	101-500
Huawei Technologies Co.,Ltd	The world's leading information and communication (ICT) infrastructure and smart terminal provider	Guangdong	September 15, 1987	4,030,813.18	>500
Sangfor Technologies Inc	Security and cloud computing solution provider	Guangdong	December 25, 2000	40,901.47	>500
Goodwill Information Technology Co., Ltd	Information system development, sales, and service provider	Beijing	July 13, 2005	51,000.00	>500
Mediinfo I.t.Co.,Ltd	Medical information service provider	Zhejiang	September 6, 1999	10,224.00	101-500
Alibaba(China)Network Technology Co., Ltd	—^b^	Zhejiang	September 9, 1999	6,942,460.80	>500
Unicom (Guangdong) Industrial internet Co., Ltd	—	Beijing	June 18, 1994	10,481,551.96	>500
Neusoft Corporation	Internet and software product and service provider	Liaoning	June 17, 1991	124,237.03	>500
Winning Health Technology Group Co., Ltd	Medical and health information solution provider	Shanghai	April 7, 2004	164,100.58	>500
China Mobile Communications Group Co., Ltd	—	Beijing	July 22, 1999	30,000,000.00	>500
Ruijie Networks Co., Ltd	Informatization solution provider, China's leading brand of data communication solutions	Fujian	October 28, 2003	50,000.00	>500
Shanghai KingYee Information Technology Co., Ltd	Smart medical technology and service provider	Shanghai	August 7, 2009	11,776.88	>500
Heren Health Co., Ltd	Medical information provider	Zhejiang	October 26, 2010	11,719.05	>500
Dnake (Xiamen) Intelligent Technology Co., Ltd	Smart hardware developer	Fujian	April 29, 2005	12,000.00	>500
Baidu Online Network Technology (Beijing) Co., Ltd	Informatization solution provider in the medical field	Beijing	January 18, 2000	29,257.96	>500
Bringspring Science and Technology Co.,Ltd	Smart city, smart medical solutions, data center integration and operation, and maintenance services, financial IT outsourcing service provider	Liaoning	February 8, 2012	59,752.79	>500
Beijing Tianjian Yuan Da Tecnology Co., Ltd	Professional developer of medical information system	Beijing	August 9, 2005	17,091.03	>500
Lianren Health and Medical Big Data Technology Co., Ltd	Medical big data analysis service provider	Shanghai	November 18, 2019	200,000.00	51-100
Wanma Technology Co., Ltd	Medical information service and hardware provider	Zhejiang	January 28, 1997	13,400.00	>500
Honeywell Integrated Technology (China) Co., Ltd	Aviation products and services, building, home, and industrial control technology, automotive products, turbochargers, and special material R&D and manufacturer	Shanghai	January 1, 1988	19,289.54	>500
NSFOCUS Technologies Group Co., Ltd	Enterprise-level information security service provider	Beijing	April 25, 2000	79,967.41	>500
Suzhou MedicalSystem Technology Co., Ltd	Comprehensive solution provider for clinical information systems and digital hospitals	Jiangsu	August 14, 2009	11,245.48	>500
Enjoyor Co.,Ltd	Mobile computing, intelligent identification, data fusion, and other technology developers	Zhejiang	November 13, 1992	65,578.91	>500
China Telecom Corporation Limited	—	Beijing	September 10, 2002	8,093,236.83	>500
Shanghai Aihui Healthy Technology Co., Ltd	Bedside information service provider	Shanghai	September 22, 2016	13,007.04	51-100
Edan Instruments, Inc	R&D, production, sales, and service provider of medical electronic equipment	Guangdong	August 2, 1995	58,172.18	>500
Nexans (Suzhou) cable solution Co., Ltd	—	Jiangsu	April 17, 2013	32,365.00	101-500
Yonyou Network Technology Co., Ltd	Data collection and business application solution provider	Beijing	January 18, 1995	324,872.13	>500
FUJIFILM (China) Investment Co., Ltd	Film R&D producer	Shanghai	April 12, 2001	21,339.70	101-500
Zhejiang Jandar Technology Co., Ltd	Software development, information system, integration service provider	Zhejiang	November 19, 1999	10,000.00	101-500
Newlink Technology Inc	—	Beijing	August 15, 2011	10,203.04	101-500
Dell (China) Company Limited	Electronic equipment manufacturer	Fujian	December 29, 1997	17,186.84	>500

^a^A currency exchange rate of RMB 1=US $0.16 is applicable.

^b^Not available.

### Analysis of Industrial Fields, Applied Technologies, and Related Concepts

The nature of the enterprises, current hot concept directions, and the essence behind it could be analyzed by the keywords of exhibitors provided by the CHINC official website and the industrial field, applied technology, and concept of the enterprises provided by the VBDATA company library.

The conference's official website provided 151 enterprise keywords, which indicated the exhibition direction of the participating enterprises. We created word frequency statistics for the keywords. The keyword “smart hospitals” appeared 41 times at most and “internet hospitals” 22 times. The rest is shown in Table 3. We drew a cloud map according to the keyword frequency.

**Figure 6:**
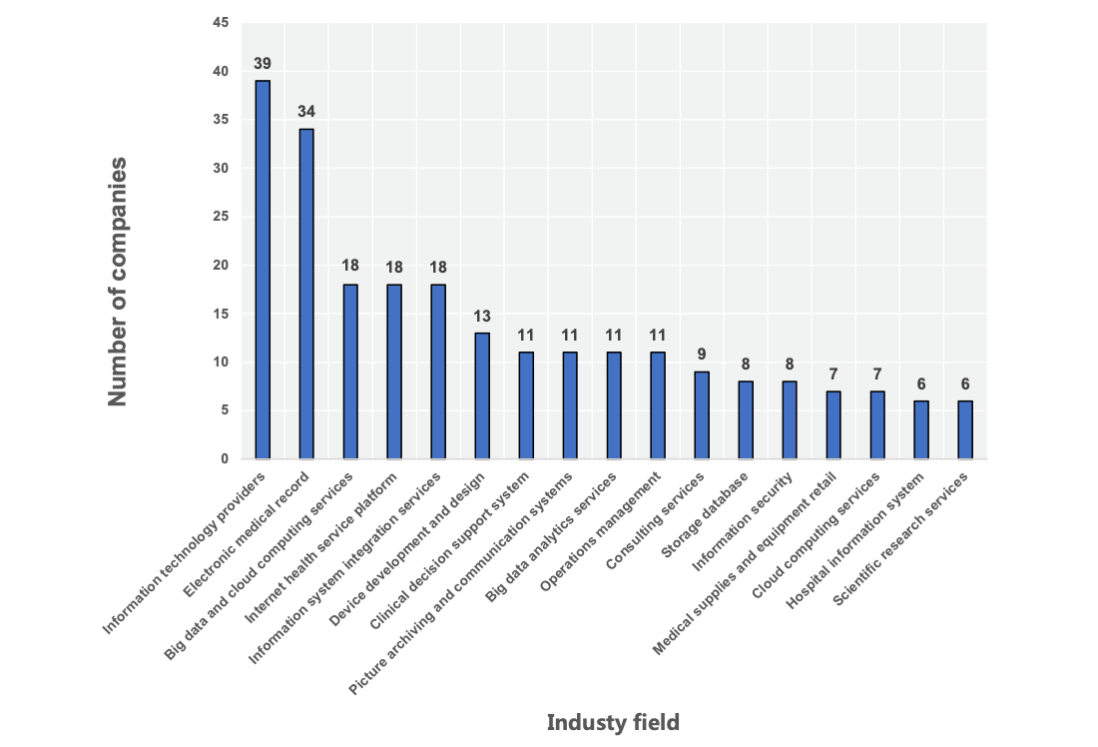
Industry field of enterprises.

**Figure 7:**
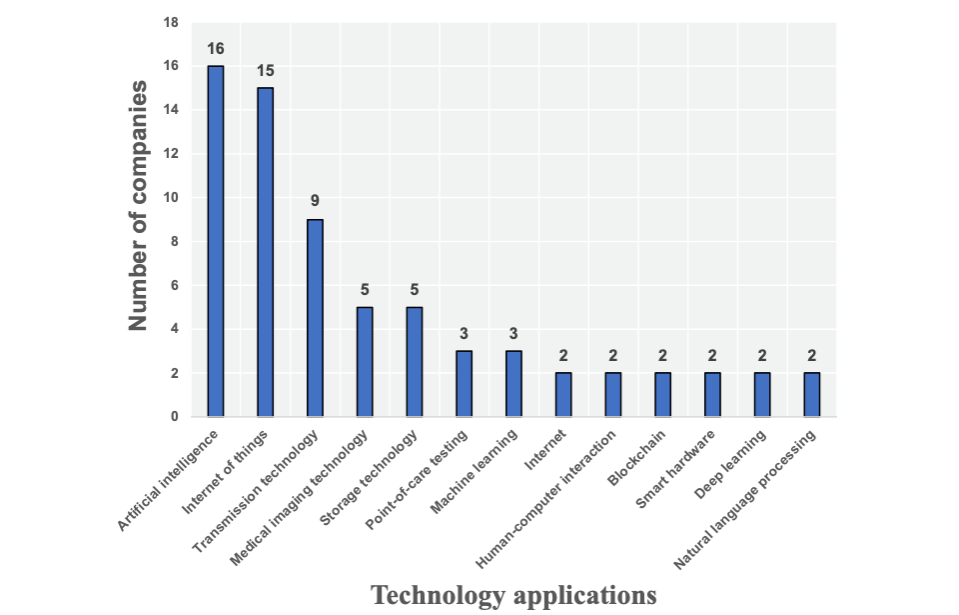
Technologies used by enterprises.

A total of 126 enterprises were found in the VBDATA company library. All (100%) companies disclosed their industrial field, 57 (45.2%) companies disclosed the technology used, and 114 (90.5%) companies provided the relevant concept labels. In the industry field, the number of information technology vendors was the largest (39/126, 30.9%), followed by EMRs (34/126, 26.9%). The distribution of industrial fields is shown in Figure 6. We also calculated statistics of the technologies used by enterprises. Of the 57 application technology enterprises, 16 (28.1%) use artificial intelligence (AI) technology and are ranked first, and 15 (26.3%) use the internet of things technology and are ranked second. The distribution of other technologies is shown in Figure 7. The VBDATA company library also provides conceptual labels of current mainstream products and innovative technologies of each enterprise, similar to the keywords given on the official CHINC website. We compared the top 15 concepts with the highest frequency by comparative analysis, and the results are shown in Table 3. Both smart medicine and internet hospitals were the focus of enterprises, and big data appeared most in VBDATA, but they were rarely mentioned at this conference.

**Table 3:** Enterprise concepts of VBDATA and enterprise keywords given by CHINC^a^.

Rank	VBDATA concept	Enterprises, n	CHINC enterprise keywords	Appearance, n
1	Big data	34	Smart hospital	41
2	Smart health care	29	Internet hospital	22
3	COVID-19	27	Smart health care	19
4	SaaS	21	Medical community	17
5	Telemedicine	14	Big data	14
6	Cloud computing	14	Electronic medical records	13
7	Internet hospital	9	Smart services	11
8	Medical equipment	9	Artificial intelligence	10
9	AI device	7	Hospital informatization	10
10	Industrial internet	7	Internet of things	10
11	Medical device supplies	6	Medical cluster	9
12	Equipment	6	Integration platform	9
13	Consumer health care	5	Hospital information system	7
14	mHealth	5	Interoperability	7
15	Public health services	5	Internet health care	7

^a^CHINC: China Hospital Information Network Conference.

## Discussion

### Preliminary Results Have Been Achieved in the Process of Hospital Informatization in China

Many information manufacturers have emerged in the past 10 years, and hospital information has basically completed the primary stage of popularization. Our research group has predicted that by 2021, the popularization rate of EMRs in domestic secondary hospitals or higher hospitals may exceed 80%. The popularization rate of EMRs in tertiary hospitals may even exceed 95% [14]. According to official Chinese documents published by the National Health Commission, the rate of tertiary public hospitals participating in grading the EMR application level in 2019 was 99.36%, which confirmed our prediction. From the perspective of enterprises, approximately half of the information-based manufacturers were established in the past 10 years, of which approximately 25% were established in the past 6 years (see the Exhibitors’ Characteristics section). With the help of innovative technologies, including AI and the internet of things (see the Analysis of Industrial Fields, Applied Technologies, and Related Concepts section), they have launched information-based solutions for various scenarios in hospitals.

It can be foreseen that the traditional basic content-related market in hospital information construction, for example, hospital information system (HIS), EMRs, laboratory information system (LIS), and picture archiving and communication system (PACS), has been gradually saturated. Therefore, the discussion of traditional hospital information systems and the exhibition of related products at this conference are not particularly ongoing. A new generation of hospital management systems began to appear in 2017, but few hospitals have the courage to carry out thorough information reform [15]. However, the National Health Commission of China has adopted the policy of linking EMR ratings with hospital performance appraisals in the past 3 years. COVID-19 will further strengthen the basic content of hospital informatization construction and gradually encourage new directions of construction. The reform will promote interconnection and high integration between information systems, provide high-quality management and treatment support by using operation data and clinical data, rebuild the hospital management system, and finally complete the digital and intelligent transformation of hospitals [16].

### Smart Hospitals Are the Focus of Hospital Construction in the Future

Under the background that the informatization of tertiary hospitals has been basically completed and is steadily being carried out in secondary hospitals, the National Health Commission issued related official documents and further released a document revision in May 2020. At present, the complete definition and construction standards of smart hospitals have not been unified at the national level, and the focus and direction in the exploration of smart hospital construction are not the same among hospitals. The government pointed out that the scope of smart hospitals mainly includes three areas: *smart medical care* for medical personnel, *smart service* for patients, and *smart management* for hospital management. However, it is certain that different from the previous informatization handing over the paper process to the computer for processing, the essence of smart hospitals is to collect, use, and analyze the data inside and outside the hospital. The purpose of smart hospitals is to provide basic support for hospital scientific research, clinical and management activities, and, finally, feedback to doctors and leaders for decision making.

Meanwhile, the government has put forward strict scoring requirements for the performance appraisal of 3-level public hospitals for several consecutive years. The performance appraisal includes data quality on the first page of EMRs, the application level of EMRs, comparability of clinical tests, the degree of intelligence of medical services, and the equality of rational use of liquid medicine. This is also an important factor in promoting the construction of smart hospitals.

The largest focus of enterprises in this conference is smart hospitals, which can be seen in Figure 6 and Table 3. Smart hospital solutions emerge one after another, mainly including the following points: First, we focus on scientific research systems and advocate data governance. Natural language processing helps the knowledge graph build a hospital special disease database and assist doctors in efficient scientific research to realize intelligent medical treatment. Related projects were carried out by Hangzhou Century Co Ltd., Shanghai Senyi Intelligent Technology Co Ltd., and Anxiang Medical Technology (Shenzhen) Co Ltd. Second, EMR quality control and diagnosis-related group (DRG)/big data diagnosis-intervention packet (DIP) are continuously hot. Automatic coding technology based on AI is introduced to promote exemplary management of hospitals and meet the quality of the first page of EMRs. Companies such as Hangzhou Firetree Technology Co Ltd. and BaseBit AI have designed intelligent operation systems. Third, the clinical decision support system (CDSS) still focuses on a single disease, and a knowledge graph is still the main technology. Deep learning methods are still used in medical image recognition. Companies such as Beijing Shenrui Bolian Technology Co Ltd, Beijing Airdoc Technology Co. Ltd., and Suzhou Mediston Medical Technology Co Ltd. are involved in this type of business. Fourth, we emphasize the ability of the middle platform and build an office automation (OA) system of smart hospitals. We also realize the personalization of different hospitals by using a middle platform and promote the office mode of a new generation of smart hospitals, on which DingTalk advocates and Xiniu Health Technology (Zhejiang) Co Ltd. is focusing. Fifth, the internet of things is hardware-upgraded to ensure intranet security and clinical efficiency. Hardware companies, such as Ruijie Networks Co Ltd. and Onco Information Technology (Shanghai) Co Ltd., have launched hospital dual-network routers based on Wi-Fi 6.0 and 5G to ensure strict internal and external network isolation, realizing intelligent wards.

The year 2021 was the first year of the 14th 5-year plan. Under the guidance of the above policies, the construction of smart hospitals has received more attention from hospitals. Starting from this demand, enterprises at conferences have launched customized smart hospital construction services.

### The Construction of a Compact County Medical Community Has Become a New Focus of Enterprises

Compared with other countries, China faces more severe challenges in the distribution of medical services. Although China has the largest number of hospitals globally, the distribution of medical resources is extremely uneven: 80% of medical resources and patients are concentrated in large hospitals and 20% in community general clinics [17]. China introduced the market mechanism into the medical service system in the 1980s, and people can go to any level of medical institutions according to their wishes. Primary medical institutions no longer play the role of health gatekeepers. Many patients give priority to higher-level medical institutions when they need medical assistance. This has led to a large reduction in patients in grassroots hospitals, a decline in the level of grassroots health service personnel [18], and a rapid increase in medical expenditure [19]. The concentration of medical resources in high-level medical institutions further weakens the ability of grassroots health services, resulting in more detours and more waste.

The Chinese government is trying to solve the uneven distribution of medical services by promoting the integration of regional health services. With the Chinese government's strong push for health care reform in 2009, the first contact point between the hierarchical medical service system and grassroots medical institutions was proposed as a key task. The reform strategy notes that the construction of a regional medical consortium is the key to promoting hierarchical diagnosis and treatment [20]. At present, the construction methods of the medical union in China mainly include the Cross-Regional Professional Alliance, the Urban Medical Group, and the County Medical Community. The importance of a compact county medical community is particularly prominent for China’s large rural population. Therefore, carrying out the integration of health services, realizing cooperation between medical institutions at all levels in rural areas, and improving the quality of health services, treatment rate, and patient satisfaction is the fundamental way to truly enable the majority of grassroots residents to obtain health-centered, equal, homogeneous, and integrated health care services [21].

Information construction is an important basis for the construction of compact county medical communities. There are obvious information system breaks between different medical institutions in county medical communities. In the past, the township-level health institution system lacked unified development and business processes, data processes lacked unified norms, and the phenomenon of *information islands* was serious [22]. County medical communities require a high degree of entity integration and information interconnection to ensure the high continuity of medical services and truly realize the original intention of common service, common interests, common responsibilities, and common development.

The market scale of medical community information construction is huge. China officially issued a document to determine Shanxi and Zhejiang Provinces as pilot provinces of the medical community and 567 counties as pilot counties in September 2019. The compact county medical community is developing rapidly, and a unified, efficient, and easy-to-use regional health information system is one of the important information supports. According to national statistics, there are 2843 county-level divisions in China, and the informatization project of the medical community in each county is approximately 40-80 million. It is roughly estimated that the market scale reaches approximately 113.7-227.4 billion RMB. A new county-level medical and health service system with clear objectives, clear rights and responsibilities, and division of labor and cooperation should be preliminarily established in 500 county-level units, gradually forming a community of services, responsibilities, interests, and management, and finally used throughout China [23]. At this conference, the county medical community informatization solution has become the struggle focus of major medical IT enterprises. Enterprises, such as YLZ Information Technology Co., Ltd., proposed establishing a regional information platform and achieving regional information interconnection, data sharing, and aggregation through unified data standards and service specifications. Big data mining and analysis technology can also be used to conduct intelligent analysis and judgment on operation management, providing intelligent auxiliary services for managers' scientific decision making.

### Whole-Course Management and the Concept of Digital Therapy Are New Hotspots and Starting Points of HIT

It is worth mentioning that with many information manufacturers in hospitals and relatively mature solutions, there is a huge potential development space for targeting the market for out-of-hospital medical services, and several new technologies and solutions have emerged. These products are mainly named after the concepts of *special disease bank*, *scientific research follow-up*, *whole-course management*, and *digital therapeutics* (DTX). It is widely recognized that most chronic diseases need comprehensive management outside the hospital and cannot be cured by short-term drugs in the hospital. At present, the best intervention measure is to carry out various self-management measures of the patients' diet, exercise, and medication outside the hospital. This requires patients to have a certain reserve of medical knowledge, to grasp their own disease changes clearly, and to have high compliance. *Digital therapy* based on emerging technologies, such as mobile medicine, big data, and AI, is the potential best solution. DTX is an intervention program driven by software programs and based on evidence-based medicine that is used to treat, manage, or prevent diseases [24]. Digital therapy transforms the existing medical principles, medical guidelines, or standard treatment schemes into application software–driven interventions by digital means, which can effectively improve the compliance and accessibility of patients' chronic disease management. It is an innovative way to overcome the limitations of traditional drug treatment [25]. Compared with the application of assisted diagnosis, telemedicine, and all new technologies in health, digital therapy can be used alone or together with other therapies to promote disease remission [26].

At this conference, we can see that several companies, such as Weimai Technology Co., Ltd. and Hangzhou Zhuojian Information Technology Co., Ltd., mentioned the concept of digital therapy and proposed corresponding solutions. However, the clinical effect of such schemes has not been verified. This lack of progress may be related to several reasons. On the one hand, the landing effect of products is poor, and the products labeled with *digital therapy* are often simple technical upgrades of traditional business products. On the other hand, most products have not been clinically verified or recognized by peers. Whole-course management and digital therapy are mostly based on concepts. The process of scientific research and standardized verification based on inquiry medicine in the clinic should be accelerated. Due to the relatively mature informatization in hospitals and the large gap and imagination space of out-of-hospital medical services, the corresponding informatization has a large development space and many opportunities in the future, which may form new hotspots.

### Conclusion

China's tertiary hospital informatization construction has basically completed the primary stage of popularization, showing two characteristics. First, China's medical information industry is focusing on the construction of smart hospitals. The most important directions include a smart system to support doctors' scientific research, a DRG smart operation system and an OA system to support hospital management, a single-disease CDSS to assist doctors in clinical practice, and the smart internet of things for logistics. Second, under the guidance of the practical needs for improving the quality of grassroots health services and national policies, the construction of a compact county medical community has become a new focus of enterprises. In addition, it can be foreseen that whole-course management and digital therapy will become new hotspots in the future. The process of scientific research and standardized verification should be accelerated.

## Abbreviations

AI: artificial intelligence
AMIA: American Medical Informatics Association
CDSS: clinical decision support system
CHINC: China Hospital Information Network Conference
CHITEC: China Health Information Technology Exchange Conference
CMIAAS: Chinese Medicine Information Association Annual Symposium
CPMI: China Proceedings of Medical Informatics
DIP: big data diagnosis-intervention packet
DRG: diagnosis-related group
DTX: digital therapeutics
EMR: electronic medical record
HIMSS: Healthcare Information and Management Systems Society
HIS: hospital information system
HIT: health information technology
LIS: laboratory information system
OA: office automation
PACS: picture archiving and communication system
